# Genome-wide identification, classification and analysis of heat shock transcription factor family in maize

**DOI:** 10.1186/1471-2164-12-76

**Published:** 2011-01-27

**Authors:** Yong-Xiang Lin, Hai-Yang Jiang, Zhang-Xin Chu, Xiu-Li Tang, Su-Wen Zhu, Bei-Jiu Cheng

**Affiliations:** 1 Key Lab of Crop Biology, School of Life Sciences, Anhui Agricultural University, Hefei, Anhui, China

## Abstract

**Background:**

Heat shock response in eukaryotes is transcriptionally regulated by conserved heat shock transcription factors (Hsfs). Hsf genes are represented by a large multigene family in plants and investigation of the Hsf gene family will serve to elucidate the mechanisms by which plants respond to stress. In recent years, reports of genome-wide structural and evolutionary analysis of the entire Hsf gene family have been generated in two model plant systems, *Arabidopsis *and rice. Maize, an important cereal crop, has represented a model plant for genetics and evolutionary research. Although some Hsf genes have been characterized in maize, analysis of the entire Hsf gene family were not completed following Maize (B73) Genome Sequencing Project.

**Results:**

A genome-wide analysis was carried out in the present study to identify all Hsfs maize genes. Due to the availability of complete maize genome sequences, 25 nonredundant Hsf genes, named *ZmHsfs *were identified. Chromosomal location, protein domain and motif organization of ZmHsfs were analyzed in maize genome. The phylogenetic relationships, gene duplications and expression profiles of *ZmHsf *genes were also presented in this study. Twenty-five ZmHsfs were classified into three major classes (class A, B, and C) according to their structural characteristics and phylogenetic comparisons, and class A was further subdivided into 10 subclasses. Moreover, phylogenetic analysis indicated that the orthologs from the three species (maize, *Arabidopsis *and rice) were distributed in all three classes, it also revealed diverse Hsf gene family expression patterns in classes and subclasses. Chromosomal/segmental duplications played a key role in Hsf gene family expansion in maize by investigation of gene duplication events. Furthermore, the transcripts of 25 *ZmHsf *genes were detected in the leaves by heat shock using quantitative real-time PCR. The result demonstrated that *ZmHsf *genes exhibit different expression levels in heat stress treatment.

**Conclusions:**

Overall, data obtained from our investigation contributes to a better understanding of the complexity of the maize Hsf gene family and provides the first step towards directing future experimentation designed to perform systematic analysis of the functions of the Hsf gene family.

## Background

All organisms possess an evolutionarily conserved, rapid cellular defense mechanism commonly designated as the heat shock (HS) response, which activates a variety of reactions in response to heat stress and a number of chemical stressors. It is characterized by rapid reprogramming of gene expression, leading to the production of a defined set of proteins called heat shock proteins (Hsps), most of which act as molecular chaperones [1,2]. At the onset of stress, Hsps prevent protein unfolding and aggregation, thereby maintaining cellular protein homeostasis, which determines critical cellular structures and functions to regulate stress response.

Hsps expression is regulated by multiple mechanisms. The central regulators of Hsps expression are heat shock transcription factors. Hsfs are the terminal components of a signal transduction chain mediating the activation of genes responsive to heat or other stress stimuli [3,4]. Under normal growth conditions, Hsf is maintained in an inert monomer state through association with molecular chaperones such as Hsp70. In response to heat shock, Hsf is converted from a transcriptional inactive monomer to active trimmer using oligomerization domains, which function as sequence-specific trimeric DNA binding proteins. Hsfs are capable of recognizing the conserved binding motifs (heat shock elements, HSEs) within the promoters of Hsf-responsive genes [5]. The consensus HSEs contain a cis-acting sequence, which consists of multiple inverted repeats of the 5'-nGAAn-3' sequence (where n is any nucleotide) [6]. At least three 5'-nGAAn-3' repeats are required for a functional HSE, and additional reiteration of the pentameric unit results in higher affinity interactions between Hsf and HSE [7].

Plant Hsfs genes have been isolated from various species [8-11]. Similar to many other transcription factors, the Hsf family has a modular structure. Despite considerable variability in size and sequence, Hsfs are structurally and functionally conserved throughout the eukaryotic kingdom. Several highly conserved domains exist in the modular structure and all characterized Hsfs have a common core structure comprised of DNA binding and oligomerization domains [4,12]. In addition, another well-defined domain is a nuclear localization signal domain (NLS). Apart from this, a C-terminal activation domain (CTAD) and a nuclear export signal (NES) are included in some Hsfs [4,13].

Close to the N-terminal, the highly structured DNA-binding domain (DBD) is the most conserved component of Hsfs, consisting of an antiparallel four-stranded β-sheet (β1-β2-β3-β4) packed against a bundle of three α-helices (H1, H2, H3). The hydrophobic core of this domain forms a helix-turn-helix (H2-T-H3) structure required for specific recognition of the HSE conserved motif [14]. An adjacent oligomerization domain (HR-A/B region) composed of hydrophobic heptad repeats is separated from the DBD domain by a flexible linker. Through hydrophobic interactions, the heptad repeats form a helical coiled-coil structure reported responsible for the trimerization of Hsfs [15]. Plant Hsf protein families fall into three classes (A, B, and C) by peculiarities of their HR-A/B regions [4]. All class A and class C Hsfs have an extended HR-A/B region due to an insertion of 21 (class A) or seven (class C) amino acid residues between the A and B of the HR-A/B region. In contrast, class B Hsfs are discriminated from class A and C by the absence of this insertion and the presence of a single, continuous heptad repeat pattern. Furthermore, the variable length of the linker between DBD domain and HR-A/B region also offer additional support for this classification (nine to 39 amino acid residues for class A, 50 to 78 amino acid residues for class B and 14 to 49 amino acid residues for class C Hsfs) [4].

Furthermore, at the C-terminus from the HR-A/B region a cluster of basic amino acids rich in arginine and lysine residues serve as a nuclear localization signal (NLS), which is required for nuclear import [16]. The NES is positioned at the C-terminus of some plant Hsfs. The overall balance of nuclear import and export processes directed by the strength and accessibility of the NLS and NES determines intracellular distribution of plant class A Hsfs [13,17]. Sequence comparison studies and functional analyses indicate that the combination of C-terminal activator motifs (AHA motifs) adjacent to a nuclear export signal (NES) represents the core of the C-terminal activation domain (CTAD) for many plant class A Hsfs [13,18]. AHA motifs are rich in aromatic (W, Y, F), hydrophobic (L, I, V) and acidic amino acid residues (D, E). On the other hand, class B and C Hsfs have no activator function of their own resulting from the lack of AHA motifs [13].

The Hsf gene family has been thoroughly characterized in *Arabidopsis *and rice [4,19], whose genomes have been sequenced. Furthermore, Hsfs have been comprehensively studied in tomato [8,17,18]. Previous study has reported several Hsf genes cloned from maize [20]. The Maize Genome Sequence Project completed full maize genome assembly (*Zea mays *L. B73) [21]. This provides an opportunity to deduce the maize Hsf gene family and infer its evolutionary history and adaptations in heat and chemical stress response mechanisms at the molecular level. In the present study, we searched for all nonredundant sets of *ZmHsf *genes and predicted their presumed structures. The results of this work provide a foundation to better understand functional and evolutionary history of the Hsf gene family in angiosperms.

## Results

### Identification and physical locations of Hsf proteins in maize

**Figure 1:**
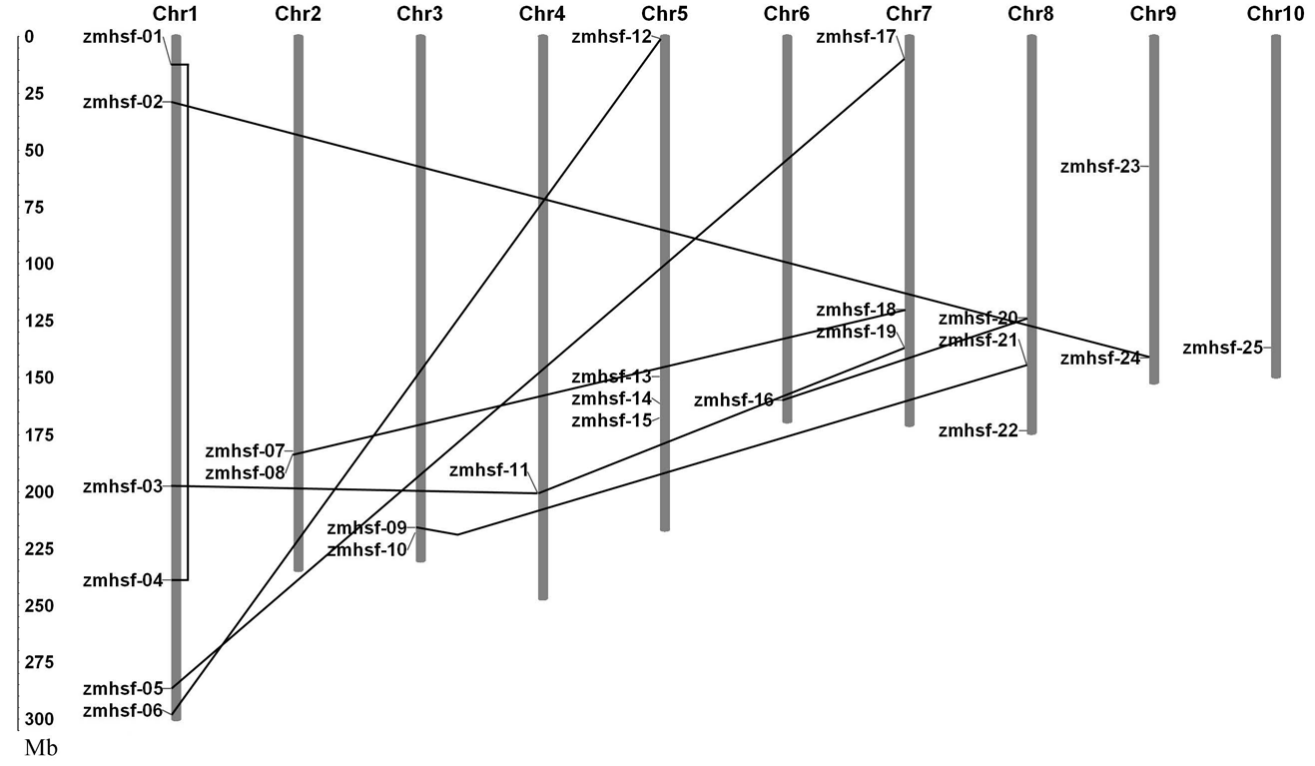
**Locations and duplications of maize Hsf paralogs on chromosomes 1-10**. The scale represents megabases (Mb). The chromosome numbers are indicated at the top of each bar.

The amino acid sequence of Hsf-type DBD domain (Pfam: PF00447) was adopted as a query in BLASTP searches for possible homologs encoded in the maize genome. As a result, 48 candidate Hsf protein sequences were identified in maize. Subsequently, all candidate Hsf protein sequences were surveyed to further verify whether they contain Hsf-type DBD domains using the Pfam database. Twenty-one candidate Hsf protein sequences were discarded for incomplete the Hsf-type DBD domain and overlapping genes. Furthermore, two sequences were removed due to the absence of a coiled-coil structure by the SMART program. Consequently, 25 nonredundant maize Hsfs were identified and described (Table 1). All nonredundant maize Hsfs were mapped on the 10 maize chromosomes (Figure 1). Hsfs were distributed in every chromosome of the maize genome, however, the number of Hsf genes on each chromosome varied widely. The largest number, comprised of six Hsf genes, was detected on chromosome 1, whereas the least number was found on chromosomes 4, 6 and 10, including only one Hsf gene.

**Table 1:** ZmHsf protein information

Number	Gene name	Translation	Size(aa)	MW (Da)	pI	Chromosome
1	*ZmHsf-01*	GRMZM2G165972_P02	384	43268.46	5.30	1
2	*ZmHsf-02*	GRMZM2G118485_P02	417	46816.21	5.09	1
3	*ZmHsf-03*	GRMZM2G164909_P01	414	44382.81	6.80	1
4	*ZmHsf-04*	GRMZM2G010871_P01	357	40502.49	4.99	1
5	*ZmHsf-05*	GRMZM2G132971_P01	359	40587.23	5.57	1
6	*ZmHsf-06*	GRMZM2G115456_P01	527	56724.50	5.11	1
7	*ZmHsf-07*	GRMZM2G088242_P01	394	41742.83	7.81	2
8	*ZmHsf-08*	GRMZM2G002131_P01	298	32270.31	9.13	2
9	*ZmHsf-09*	GRMZM2G089525_P01	331	35883.69	5.94	3
10	*ZmHsf-10*	GRMZM2G005815_P01	462	50970.67	8.87	3
11	*ZmHsf-11*	GRMZM2G098696_P01	370	39562.03	5.89	4
12	*ZmHsf-12*	GRMZM2G384339_P01	497	54104.58	5.06	5
13	*ZmHsf-13*	GRMZM2G105348_P01	257	27836.98	5.85	5
14	*ZmHsf-14*	GRMZM2G179802_P02	528	58138.65	5.57	5
15	*ZmHsf-15*	GRMZM2G059851_P01	508	56061.66	4.96	5
16	*ZmHsf-16*	AC206165.3_FGP007	469	51623.79	5.41	6
17	*ZmHsf-17*	GRMZM2G125969_P01	375	42043.73	4.70	7
18	*ZmHsf-18*	GRMZM2G139535_P01	298	32258.35	9.53	7
19	*ZmHsf-19*	GRMZM2G165272_P01	394	41468.10	5.00	7
20	*ZmHsf-20*	AC205471.4_FGP003	446	49718.46	5.15	8
21	*ZmHsf-21*	GRMZM2G086880_P01	348	37409.50	8.09	8
22	*ZmHsf-22*	GRMZM2G118453_P01	433	48647.71	5.25	8
23	*ZmHsf-23*	GRMZM2G173090_P02	350	38154.66	4.95	9
24	*ZmHsf-24*	GRMZM2G026742_P01	407	45317.63	4.97	9
25	*ZmHsf-25*	GRMZM2G301485_P01	318	33947.51	5.70	10

Heat shock transcription factors identified from maize are listed with their names according to their chromosome location in Figure 1. Proteins are designated according to their Sequenced ID, the protein sequence length, molecular weight (MW), isoelectric point (pI) and chromosomal locations.

### Conserved Domains and Motifs in maize Hsf proteins

**Figure 2:**
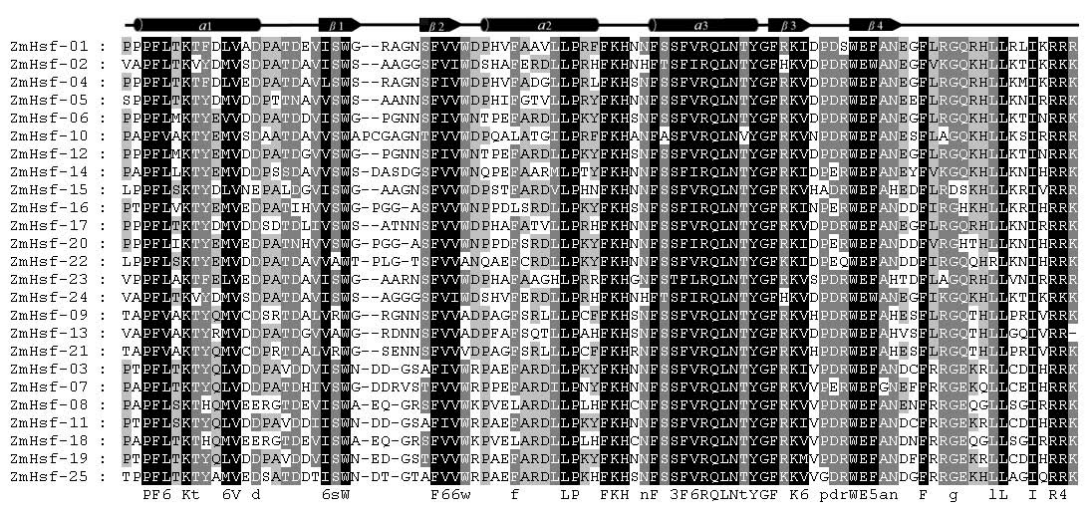
**Multiple sequence alignment of the DBD domains of the Hsf protein family in maize**. The definition of the Hsf numbers corresponds to the order of alignment. The multiple alignment results clearly show the highly conserved DBD domains among maize Hsf genes. The secondary structure elements of DBD (α1-β1-β2-α2-α3-β3-β4) are shown above the alignment. Cylindrical tubes represent α-helices and block arrows represent β-sheets.

**Figure 3:**
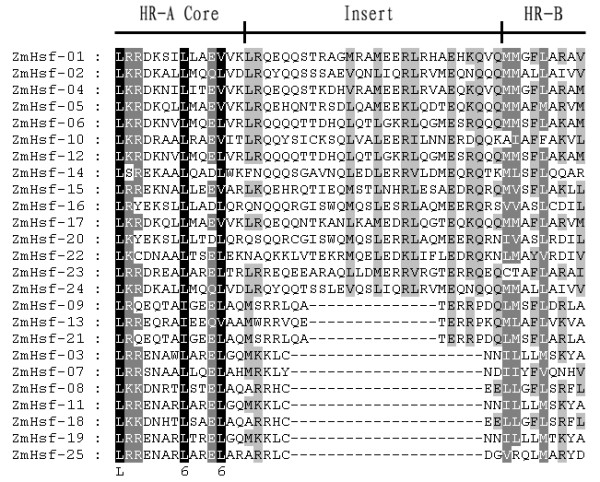
**Multiple sequence alignment of the HR-A/B regions of the Hsf protein family in maize**. The definition of the Hsf numbers corresponds to the order of alignment. The scheme at the top depicts the locations and boundaries of the HR-A core, insert, and HR-B regions within the HR-A/B regions.

The modular structure of heat shock transcription factor was studied thoroughly in some model plants [4,8]. The detailed knowledge regarding tomato and *Arabidopsis *Hsfs functional domains enabled us to analyze similar domains for the 25 Hsfs identified from the maize genome (Table 2). Five conserved domains were observed in the majority of the maize Hsf proteins. The multiple alignment clearly showed the highly structured DBD domain of approximately 100 amino acids, located in the proteins amino-terminal section, which was the most conserved section of maize Hsfs (Figure 2). MARCOIL was used to predict the coiled-coil structure characteristic of Leu-zipper type protein interaction domains, which is a property of the HR-A/B region in the Hsf protein sequences. The putative HR-A/B regions were consistently characterized by the predicted coiled-coil structure (Figure 3). Information regarding the potential NLS and NES domains in maize Hsf protein sequences, which are crucial for dynamic intracellular distribution of Hsfs between the nucleus and cytoplasm, were obtained by PredictNLS and NetNES. Nearly all Hsfs contain two clusters of basic amino acid residues (K/R motifs), which might serve as potential NLS motifs. Results obtained from mutation analysis of the two potential NLS motifs from two related tomato Hsfs (HsfA1 and HsfA2) indicated that only one of the two motifs adjacent to the HR-A/B region, and not the conserved C-terminal part of the DBD domain is functional as an Hsf protein nuclear localization signal [16,17,22]. Prediction programs, sequence comparisons and cognitive models generated from previous research detected a wide range of putative NLSs, which were monopartite or bipartite clusters and found close to the C-terminal of the HR-A/B region of maize Hsfs. Similarly, some putative NESs were identified close to the C-terminal of maize Hsfs. However, the following exceptions were observed: ZmHsf-08, ZmHsf-18 and ZmHsf-22. ZmHsf-08 and ZmHsf-18 NESs were closer to the HR-A/B regions than to NLSs. In particular, ZmHsf-22 NES was located in the HR-A/B domain region. As described by Nover *et al *(2001) and based on preceding investigations with the AHA motifs of tomato Hsf A1 and A2, we used sequence comparisons and predicted the putative AHA motifs in the center of the C-terminal activation domains for most class A maize Hsfs (Table 2). However, we were unable to predict class B and C putative AHA motifs.

**Table 2:** Functional domains and motifs of maize Hsfs

Gene name	Group	DBD	OD	NLS	NES	AHA motifs
*ZmHsf-01*	A2	41-134	167-217	(247) RKRRR	(296) LENLALNI	AHA (336) DDFWEELLNE
*ZmHsf-02*	A9	48-140	180-230	(254) NRKRR		
*ZmHsf-04*	A2	43-136	168-218	(233) KRK7KKRRR	(344) LAQQLGYL	AHA1 (267) LKMFESGVLN
						AHA2 (314) DDFWAELLVE
*ZmHsf-05*	A2	37-130	159-209	(224) MRK7KKRRRR		AHA (314) DDFWEDLLHE
*ZmHsf-06*	A1	56-149	186-236	(261) KKRR	(505) IGDLTEQM	AHA (470) DSFWEQFL
*ZmHsf-10*	A6	137-232	263-313	(337) KRQR		AHA (401) SDVWDELDLD
*ZmHsf-12*	A1	29-122	159-209	(234) KKRR	(477) LTEQM	AHA (436) NSIWEQFL
*ZmHsf-14*	A5	66-160	187-237	(261) HKKRR	(387) LNLSL	AHA (479) DKFWEQFLTE
*ZmHsf-15*	A3	73-166	193-243	(267) KRKFLK	(405) LSPLPDNMG	AHA1 (432) EQIWGVDASA
						AHA2 (472) ERFWELDFQA
*ZmHsf-16*	A4	22-115	145-195	(215) SKKRR		AHA (408) DVFWERFLTD
*ZmHsf-17*	A2	49-142	172-222	(237) MRK7KKRRRR	(359) LSEKMGYL	AHA (332) DNFWEQLLNE
*ZmHsf-20*	A4	10-103	133-183	(203) SKKRR		AHA (387) DVFWERFLTD
*ZmHsf-22*	A4	7-100	130-180	(197) GKKRR	(155) MQELEDKLIF	AHA (368) DGFWQQFLTE
*ZmHsf-23*	A6	48-141	167-217	(239) RKRR	(257) LDIEELAM	AHA1 (291) DMIWYELLGE
						AHA2 (324) AQPWAEMDE
*ZmHsf-24*	A9	48-141	180-230	(254) NRKRR		
*ZmHsf-03*	B	69-162	231-260	(337) RKRMR		
*ZmHsf-07*	B	19-113	226-255	(276) RKK	(369) LALECAGLSL	
*ZmHsf-08*	B	15-108	175-203	(259) RKRAR	(206) VRQLDLGL	
*ZmHsf-11*	B	34-127	199-228	(297) RKRMR		
*ZmHsf-18*	B	17-110	172-201	(259) RKRGR	(203) VRQLDLRL	
*ZmHsf-19*	B	42-135	217-246	(318) KRMR		
*ZmHsf-25*	B	8-101	161-190	(232) KRLR	(279) LDVLTLSV	
*ZmHsf-09*	C	24-117	171-207	(224) KKRRR		
*ZmHsf-13*	C	12-104	135-171	(202) KRAR		
*ZmHsf-21*	C	32-125	176-212	(229) KKRRR		

Numbers in brackets indicate position of the putative nuclear localization signal (NLS), nuclear export signal (NES), and activator (AHA) motifs in the C-terminal domain. Putative NLS can be monopartite (e.g., ZmHsf-01, ZmHsf-09) or bipartite (e.g., ZmHsf-04, ZmHsf-05).

**Figure 4:**
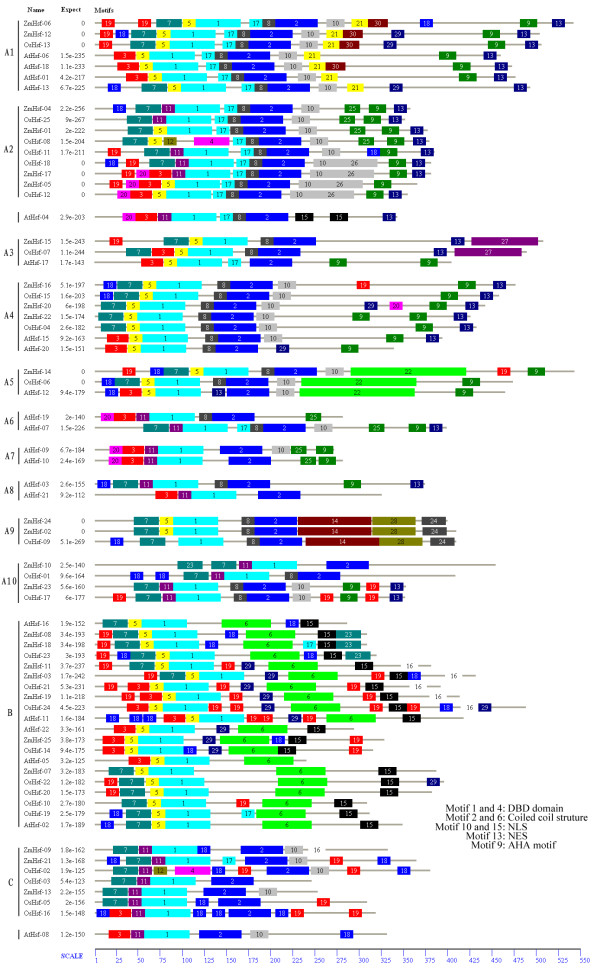
**Distribution of conserved motifs in the Hsf family members**. All motifs were identified by MEME using the complete amino acid sequences of 71 maize, rice and *Arabidopsis *Hsf genes documented in Figure 5. Names of all members among the defined gene clusters and combined *P*-values are shown on the left side of the figure and motif sizes are indicated at the bottom of the figure. Different motifs are indicated by different colors numbered 1-30. The same number in different proteins refers to the same motif with the exception of motif 15 in AtHsf-04. Because AtHsf-04 exhibits two boxes of motif 15, the box near the N-terminal represents NLS and the box close to the C-terminal specifies AHA. For details of motifs refer to Table 3.

MEME web server was employed as a secondary method to analyze motif distribution and verify the results of domain prediction (Figure 4; Table 3). Specifying the DBD domain, motif 1 was found in 25 members of the maize Hsf family. Specifying the coiled-coil structure, motifs 2 and 6 were distinctively detected in all members of maize Hsf family. All class B proteins exhibited the motif 6-type coiled-coil region, whereas motif 2-type coiled-coil region was only detected in classes A and C. The conserved motifs 10 and 15 were identified as NLS, which were widely distributed in the maize Hsf family. Motif 10 was characteristic of class A and class C, and the NLS domain was represented by motif 15 in class B. Furthermore, motifs 13 and 9 represented NES and AHA motifs, respectively, which were detected close to the Hsfs C-terminal. Lastly, some unknown motifs were also identified by MEME motif analysis.

**Table 3:** Motif sequences identified by MEME tools

Motif	Multilevel consensus sequence
1	PKYFKHNNFSSFVRQLNTYGFRKIDPDRWEFANECFLRGQKHLLKNIHRRKP
2	NVLMQEVVKLRQQQQTTKWQMQAMEQRLQHMEQRQQQMMSFLAKVMQNP
3	PFLTKTYDMVDDPATDHVISWNEDN
4	SFVRQLNTYGFRKVDPDRWEFANECFLRGQKHLLCNIHRRK
5	SFVVWNPHEFARDLL
6	AGSCPAYADLMEENERLRRENARLTRELAHMKKLCNNIIYFMSNYVDPQQPDAAKAM
7	SGPAPFLTKTYQMVCDPATDHVISWGPCG
8	YFGYEEEIERLKRDK
9	AEINDDFWEQFLTEGPGCCE
10	EMRKELIDAISKKRRRPIDDC
11	SFVVWDPHAFSQTLL
12	YFKHNNFS
13	DNMDVLTEQMGYLSS
14	FLNQLVQQQRRSNWWNDDGNRKRRFQALEHGPVDDQETSGGGAQIIQYCPPVPETSNQPIPANEAFCSTPAQPVSSPALEMPMDV
15	DEDKCVKLFGVSIGDKRMRDH
16	QPWPIYRPRPVYHPIRPCNG
17	PQYQQQSVGSCVEVG
18	GGGGGGGG
19	AAAAAA
20	PMEGLHEVGPP
21	ASLDGQIVKYQPMINEAAK
22	TSFYDDHSSTSKQEMGNLLNQHFSDKLKLGLCPAMTESNIITLSTQSSHEDNGSPHGKHPDCDMMGMECLPLVPQMMELSDTGTSICPSKSVCFTPPINDDGFLPCHLNLTLASCPMDVDKSQIPDANGNTID
23	RCEEAAASERPIKMIRIGEPWIGVPSSGP
24	YDHPWLEQDCQMEAQQNCKNPQYADVIT
25	ELENLALNIQGLGKGKID
26	PEADDMGTGSSLEQGSPVLFEPQDPVEFLIDGIPSDLESSAVDAHGLIAPQDI
27	MDADDDERIWGVDASAALQSSCSGTSQQAYGSHVSDPYLMDIANKPEKFWELDFQALDDGDLQLDKCVIDDPALQQQ
28	MASNNVGTFDSTGNDFTDTSALCEWDDMDIFGGELEHILQQPEQDFQVDP
29	SPTYSGEEQVISSNS
30	AMLRKILKLDSSHRFESMGNSDN

Numbers correspond to the motifs described in Figure 4.

Overall, despite variability in size and sequence, the predicted Hsf DBD, HR-A/B region and NLS domain were observed in each maize Hsfs by two combined methods. Although MEME motifs did not correspond precisely to individual putative NES and AHA domains defined by domain prediction of the first method, it was clearly indicated that a fraction of maize Hsfs contained NES domains and the majority of class A maize Hsfs had putative AHA domains.

### Phylogenetic and evolutionary analysis in maize Hsf proteins

**Figure 5:**
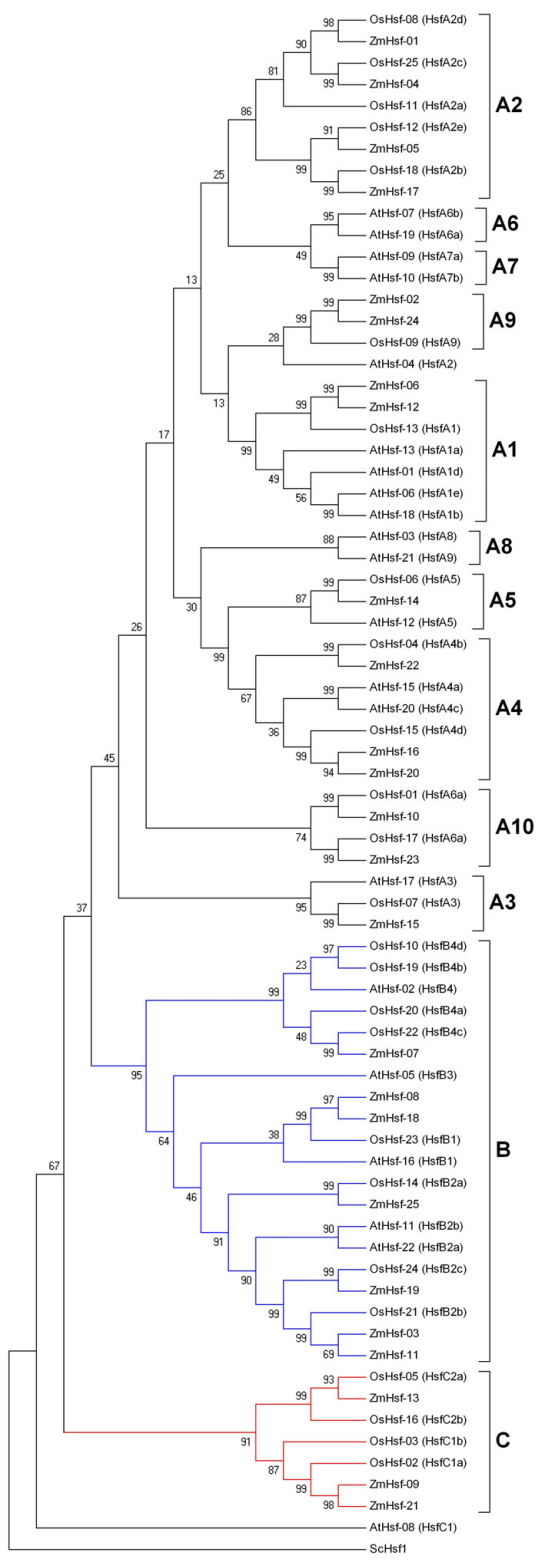
**Neighbor-joining phylogenetic tree of the Hsf members**. The phylogenetic tree, constructed with MEGA4.0, has been generated on the basis of the amino acid sequences of the N-terminal domains of Hsfs including the DNA-binding domain, the HR-A/B region and parts of the linker between both two regions. ScHsf1 was set as the outgroup. Hsf proteins are divided into twelve groups (A1, A2, A3, A4, A5, A6, A7, A8, A9, A10, B, C) based on previous research [4] and high bootstrap values (>50). The contents in brackets are corresponding Hsf annotations. Branches of members belonging to class A subclasses are represented by black lines, branches of members belonging to class B are represented with blue lines, and branches of members belonging to class C are represented with red lines. The abbreviations of species names are as follows: Zm, *Zea mays*; Os, *Oryza*; At, *Arabidopsis thaliana*.

In order to analyze the phylogenetic organization of the Hsf families, a phylogenetic analysis of 25 maize Hsfs, 25 rice Hsfs (OsHsfs) and 21 *Arabidopsis *Hsfs (AtHsfs) was performed by generating a phylogenetic tree (Figure 5). The OsHsf and AtHsf protein sequences were downloaded from the rice genome annotation (TIGR) [23] and the *Arabidopsis *Information Resources (TAIR) [24]. All Hsfs fell broadly into three major classes: classes A, B and C, with well-supported bootstrap values, which included representative genes of maize, rice and *Arabidopsis *besides AtHsf-08 (HsfC1). In this study, class A was further subdivided into ten subclasses according to their bootstrap values and phylogenetic relationship, designated as A1, A2, A3, A4, A5, A6, A7, A8, A9 and A10. In our analysis, AtHsf-21 (A9 by annotation) did not cluster with subclass A9 but was grouped into subclass A8. In addition, AtHsf-04 (A2 by annotation) and AtHsf-08 (C1 by annotation) were not classed in the Hsf subclass A2 and class C respectively. Moreover, ZmHsf-10, ZmHsf-23, OsHsf-01 and OsHsf-17 constituted subclass A10 clade. The motif distribution analyzed by MEME was also indicated in Figure 4, which was basically consistent with the phylogenetic analysis. The members of the same subclass usually share several class- and group-specific conserved motifs in addition to the Hsf DBD, HR-A/B region, and NLS domain. Putative orthologous proteins were also identified in the tree, including, for example, ZmHsf-17 and OsHsf-18, ZmHsf-07 and OsHsf-22.

### Hsf gene duplications in the maize genome

The potential mechanisms involved in the evolution of the maize Hsf gene family were elucidated by analyzing the duplication events that may have occurred during maize genome evolution. Nine total duplicated gene pairs of the 25 maize heat shock factors were identified, including eight segmental duplication events between chromosomes (e.g. *ZmHsf-08 *and *ZmHsf-18*, *ZmHsf-09 *and *ZmHsf-21*) as well as one duplication event within the same chromosome (*ZmHsf-01 *and *ZmHsf-04*) linked with lines (Figure 1). *ZmHsf-11 *participated in two duplication events with *ZmHsf-03 *and *ZmHsf-19 *and each of these three genes belonged to class B. Chromosome 10 was not involved in any duplication events.

### Digital expression analysis: EST expression profile

Maize Hsfs expression patterns were studied using corresponding EST database with known *ZmHsfs *coding sequence, resulted in the assignment of *ZmHsfs *to ten groups on the basis of tissue and organ types (Table 4). In addition, other expression evidence was verified in MAGI and PlantGDB databases (Table 4). After integrating and analyzing all expression data, we found all *ZmHsfs *were supported by expression evidence with the exception of the *ZmHsf-16 *gene. Interestingly, *ZmHsf-03*, *ZmHsf-11*, *ZmHsf-15*, and *ZmHsf-19 *were found for expression in seeds, *ZmHsf-07 *in shoot tips, and *ZmHsf-17 *in roots. Furthermore, *ZmHsf *duplicated gene pair expression patterns were investigated, only two pairs (*ZmHsf-03 *and *ZmHsf-11*, *ZmHsf-11 *and *ZmHsf-19*) of nine shared the same expression patterns between the two members of each gene pair. In the other seven duplicated gene pairs, two paralogs of each gene pair exhibited dissimilar expression patterns. *ZmHsf-04 *was detected in husks and seeds, however, its paralogue gene *ZmHsf-01 *appeared to have no tissue-specific expression pattern.

**Table 4:** Digital expression analysis of *ZmHsf *genes

**Gene**	**Tissue and organ type (NCBI)**										**Number of ESTs in dbEST**	**MAGI**	**PlantGDB**		
				
	**Silks**	**Husks**	**Shoot tips**	**Leaf**	**Root**	**Seedling**	**Tassel**	**Ear**	**Seed**	**Multiple**			**EST**	**cDNA**	**PUTs**
*ZmHsf-01*										+	9	+	+		+
*ZmHsf-02*	+		+	+		+			+	+	7	+	+		
*ZmHsf-03*									+		1		+		
*ZmHsf-04*		+							+	+	14	+	+	+	+
*ZmHsf-05*				+	+			+	+	+	20	+	+	+	+
*ZmHsf-06*	+	+		+	+	+			+	+	17	+	+	+	+
*ZmHsf-07*			+							+	3				
*ZmHsf-08*					+				+	+	27		+		
*ZmHsf-09*										+	5				
*ZmHsf-10*										+	6		+		
*ZmHsf-11*									+	+	9		+		
*ZmHsf-12*				+			+	+	+	+	11	+	+	+	
*ZmHsf-13*					+				+	+	11	+	+		
*ZmHsf-14*	+	+		+	+		+		+	+	23				
*ZmHsf-15*									+	+	6	+	+		
*ZmHsf-16*											0				
*ZmHsf-17*					+					+	11	+	+		
*ZmHsf-18*											0		+		
*ZmHsf-19*									+	+	5		+		+
*ZmHsf-20*			+					+		+	4				
*ZmHsf-21*			+	+					+		1				
*ZmHsf-22*										+	11	+	+	+	+
*ZmHsf-23*										+	2				
*ZmHsf-24*										+	8	+	+	+	+
*ZmHsf-25*										+	4		+		

+: Expressed; blank: not expressed.

### Expression analysis of *ZmHsf *gene family under heat stress treatment

**Figure 6:**
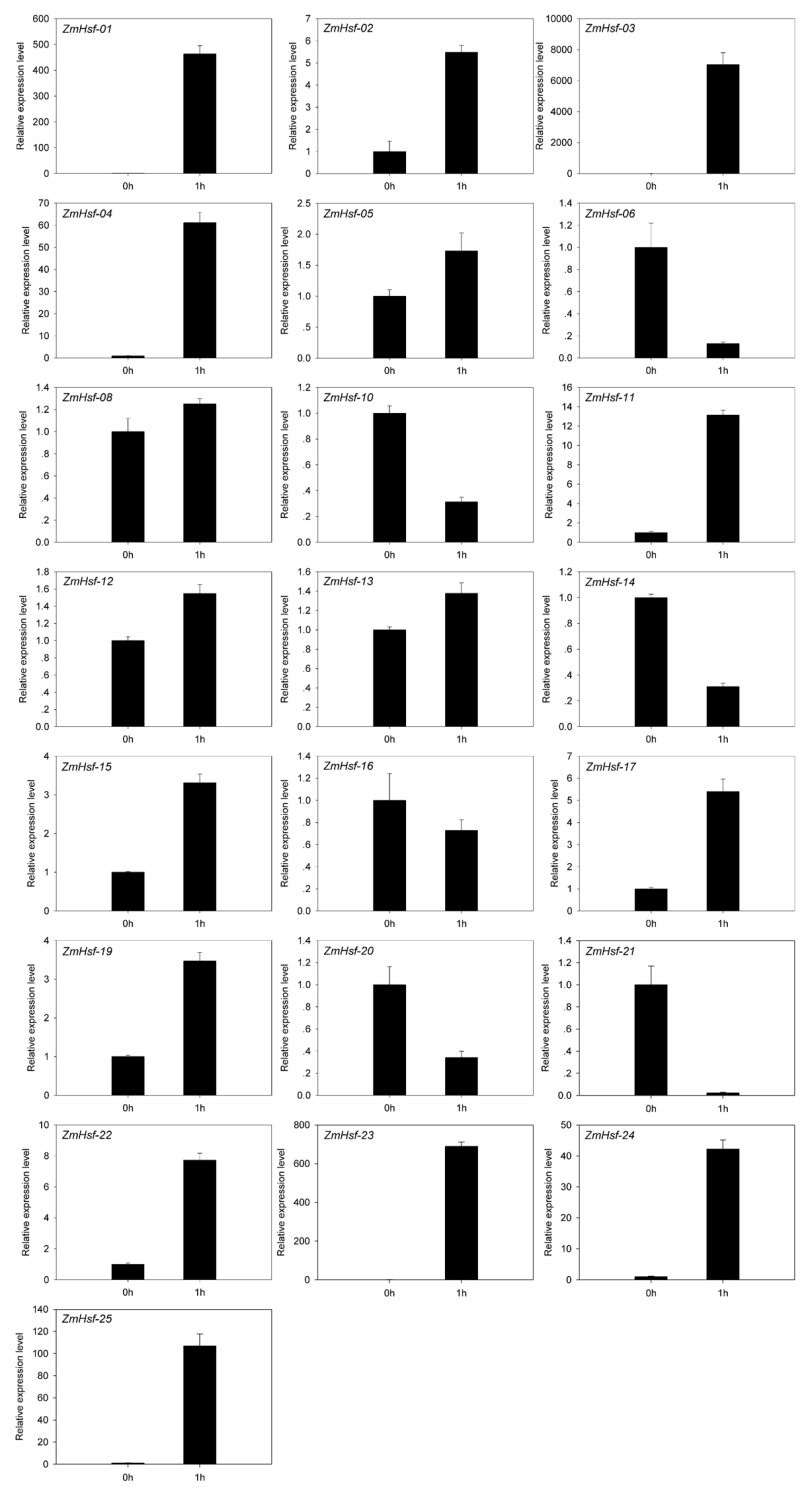
**Relative gene expression of *ZmHsfs *analyzed by qRT-PCR responsed to heat stress treatment**. qRT-PCR data was normalized using maize *Actin 1 *gene and are shown relative to 0 h. *X*-axes are time course (0 h and 1 h) and *y*-axes are scales of relative expression level (error bars indicate SD).

To examine if these predicted genes were expressed in maize and to further confirm their stress-responsiveness to abiotic stress, quantitative real-time PCR was performed for 25 *ZmHsf *genes in the leaves of maize exposed to heat stress. The analysis revealed that these genes are differentially expressed in the leaves under heat stress condition (Figure 6). Twenty-two genes showed expression activity in maize leaves by heat stress. Most of these responsive genes showed up-regulation of their expression, in which 12 genes were significantly up-regulated (>2-fold). Interestingly, six members including *ZmHsf-01*, *ZmHsf-03*, *ZmHsf-04*, *ZmHsf-23*, *ZmHsf-24 *and *ZmHsf-25 *showed no expression or only faint expression in the leaves of maize under normal growth conditions, however, they were strongly up-regulated during heat stress treatment. Five genes (*ZmHsf-06*, *ZmHsf-10*, *ZmHsf-14*, *ZmHsf-20 *and *ZmHsf-21*) were greatly down-regulated (<0.5-fold) during the heat stress treatment. Three genes (*ZmHsf-07*, *ZmHsf-09*, and *ZmHsf-18*) of 25 genes exhibited no expression in the leaves of normal and treated plants. Moreover, our results showed that the transcript levels of five *ZmHsfs *(*ZmHsf-05*, *ZmHsf-08*, *ZmHsf-12*, *ZmHsf-13 *and *ZmHsf-16*) did not present many changes in heat stress treatment. By comparing the expression data of each pair of duplicated *ZmHsf *genes, nine pairs of duplicated genes exhibited significant divergence in their expression levels following heat stress treatment. For example, *ZmHsf-06 *was dramatically down-regulated, while *ZmHsf-12 *showed slight increase in transcripts at heat stress treatment.

## Discussion

In this study, a comprehensive set of 25 nonredundant heat shock factors were identified and characterized from the current version of the maize (B73) genome. In a former publication, 22 maize Hsf isoforms were reported, which were composed of 16 Hsfs having intact ORFs and six 5' truncated Hsfs [25]. The following are likely responsible for these discrepancies. In the previous studies, the maize genome had not been completely sequenced, 22 maize Hsf genes were identified by searching the publicly available maize EST and genomic sequence survey (GSS) databases for homology to rice Hsfs. In our study, the maize genome has been completely sequenced, therefore the maize genome database used here is more precise and complete than what was previously available.

Although the maize genome is approximately 6-fold larger than rice (2,300 Mb:389 Mb), the gene number is similar (3,2000:3,7000 ) and their genetic map organization is highly conserved. We found maize and rice have the same number of Hsfs [19]. This partially accounts for the support of Hsfs conservation in these two species during the evolutionary process. In the investigation of conserved Hsf domains, we observed two class A Hsfs (ZmHsf-02, ZmHsf-24) lacking the AHA motif, which is essential for class A Hsfs transcription activity. Previous study suggests [19] these proteins bind to other class A Hsfs forming hetero-oligomers to achieve their functions.

Phylogenetic analysis of Hsfs in maize, rice and *Arabidopsis *indicated that ZmHsfs are more closely allied with OsHsfs than AtHsfs, consistent with the evolutionary relationships among maize, rice and *Arabidopsis *i.e. two monocots in the Poaceae Subclass Commelinidae and one dicot in the Brassicaceae Subclass Dilleniidae. The fact that all three classes (A, B and C) identified in maize, rice and *Arabidopsis *genes implies that the Hsf genes originated prior to the divergence of monocots and dicots. Hsfs of rice and maize appear more close relationship between each other in subclass A1 than to Hsfs from *Arabidopsis*. Such observations suggest the expansion of these Hsf genes following divergence of monocots and dicots.

The phylogenetic analysis showed that AtHsf-04 (HsfA2 type) and AtHsf-08 (HsfC1 type) were not grouped into subclass A2 and class C, respectively, and subclass A2 and class C were OsHsfs and ZmHsfs clusters. ZmHsfs and OsHsfs belong to the same clade, indicating that Hsfs of these subclasses expanded in a species-specific manner from common ancestral genes that were present prior to diversification of the monocot and dicot lineages. Phylogenetic data also proposed that subclass A2 and class C Hsfs were expanded in monocots but not in *Arabidopsis*. A single HsfA2 (AtHsf-04) is present in *Arabidopsis*. However, maize has four members and rice contains five in subclass A2. Class C consists of three maize and four rice members, while *Arabidopsis *has only one class C type member (AtHsf-08).

In addition, possible gene loss during the course of evolution was supported by phylogenetic reconstruction. Subclasses A7 and A8 exhibit interesting characteristics that monocots were not found in these two subclasses. Accordingly, this might indicates two dicot specific gene subclasses. Gene duplication events play a significant role in the amplification of gene family members in the genome [26,27]. Research has estimated the fraction of retained paralogs is 72% in maize, having occurred over the course of 11 million years of evolution [28]. The expansion mechanism of the maize Hsf gene family was analyzed to understand gene duplication events. Nine pairs of maize Hsf gene paralogs were identified. Among the paralogs, only one pair is involved in regional duplication in chromosome 1, however, two members in each of the other eight pairs were arranged between chromosomes. This result suggested the maize Hsf gene family expansion originated in a high number of large segmental duplications. An increase in the number of gene regulators (i.e. transcriptional and developmental regulators and signal transducers) is an essential factor in the evolution of more complex systems in different species [29]. It is hard to achieve the expansions of these regulator gene classes only through single-gene duplications, which points to the importance of genome duplications in expanding the regulatory gene repertoire [30]. It was estimated that more than 90% increase in regulatory genes had been caused by genome duplications in the *Arabidopsis *lineage in the last approximately 150 million years [27]. Similarly, individual gene family expansion follows this rule. In plants, genome duplications have mainly contributed to expression of the Aux/IAA family of auxin response regulators [31]. Data from studies of the maize genome revealed that its genome has experienced two rounds of genome duplications, an ancient duplication prior to the maize-rice divergence and a recent event following triploidization [32]. The association of Hsf gene expansion in maize with these two rounds of maize genome duplication explains this observation and in addition sheds light on the evolutionary process of the maize Hsf gene family. Furthermore, segmental duplications occur more often in more slowly evolving gene families, e.g. MYB gene family [26]. Due to the major role of segmental duplications in the Hsf gene family evolution, the maize Hsf gene family might hold a slow evolutionary rate.

Several approaches were employed for maize Hsf gene expression analysis by EST database. *ZmHsf *genes exhibited distinct expression patterns in different tissues or organs. One explanation is that *ZmHsf *genes have different expression patterns in various tissues and at multiple developmental stages. Expression profiles of 12 class A rice heat shock transcription factor genes have been resolved and the *OsHsfA *genes displayed tissue-specific expression under normal conditions [33]. *AtHsfA9 *was exclusively expressed during the late seed development stage and controlled by the seed-specific transcription factor abscisic acid-insensitive 3 (ABI3) [34]. Furthermore, the expression data revealed that the majority of duplicated *ZmHsf *gene pairs exhibited diverse expression patterns between two members. It suggested that functional diversification of the surviving duplicated genes is a major feature of the long-term evolution [35].

Expression analysis of quantitative RT-PCR showed that maize Hsf genes exist different expression levels by heat stress. In this study, we have detected three HsfA2-type *ZmHsfs *(*ZmHsf-01*, *ZmHsf-04 *and *ZmHsf-17*) with significantly higher expression, when subjected to heat stress. The result indicated that the *ZmHsfA2 *subclass was closely related with maize heat shock response. Moreover, six genes were remarkably up-regulated under heat stress condition, i.e. *ZmHsf-01*, *ZmHsf-03 *and *ZmHsf-23*, and *et al*., which suggested specific roles for these genes in maize during heat stress. It is noteworthy that three *ZmHsfs *(*ZmHsf-03*, *ZmHsf-11 *and *ZmHsf-25*) assigned to class B appeared to be strongly induced by heat stress. The Hsfs belong to class B lack certain structural features of the class A activator Hsfs. Class B-Hsfs may serve as transcriptional repressors or coactivator cooperating with class A Hsfs. But the functional roles of these three Class B-Hsfs in maize will require further investigations. It is likely that the *Hsf *genes remaining unaltered or down-regulated in expression may locate at downstream in the hierarchy of the events involved in heat shock response or are repressed by other members of the family [36]. In addition, if Hsp proteins accumulate enough, they may be involved in feedback regulation to repress Hsfs activity, such as Hsp70 proteins. In the nine duplicated gene pairs of maize, the significant divergence of expression levels between the two members of each gene pair implied that duplicated genes had various functions in the response to heat stress in the evolutionary history.

## Conclusions

This survey presents a comprehensive overview of the Hsf gene family repertoire within the maize draft genome. Based on structural characteristics and a comparison of the phylogenetic relationships among maize, rice, and *Arabidopsis*, all 25 ZmHsfs fell into three major classes (class A, B, C), and class A was organized into 10 subclasses. Further phylogenetic analysis revealed divergent expansion patterns of Hsf gene families in classes and subclasses. Our analyses suggest that whole genome and chromosomal segment duplications largely contributed to Hsf gene family expansion in maize. Our computational expression analyses suggest that many maize Hsf genes play functional developmental roles in multiple tissues. Furthermore, expression profiles by quantitative real-time PCR revealed that the majority of identified *ZmHsfs *most likely are expressed in maize and these genes are induced by heat stress with differential induction levels in leaves. Overall, our study will serve to better understand the complexity of the maize Hsf gene family and guide future experimental work. Together with the availability of the complete maize genome sequence and the increasing ease of obtaining mutants and raising transgenics, our analysis should facilitate functional characterization studies to confirm maize Hsfs and deduce Hsfs gene roles in plant stress responses.

## Methods

### Identification and physical locations of Hsf proteins in maize

The maize genome sequence has been completed, and filtered protein and cds sequences have also become available [21]. Initially, due to the variation in Hsf sequences, nine protein sequences known as Hsf were used to search the Pfam (*P*rotein *family*) database [37]. In this way, integrated and exact conserved Hsf-type DBD domain sequence based on the Hidden Markov Model (HMM) would be obtained. The nine query sequences were as follows: maize Hsf sequences [NCBI: ACG33027.1, ACG29285.1, ACG28818.1], rice Hsf sequences LOC_Os10g28340 (class A), LOC_Os04g48030 (class B), and LOC_Os01g43590 (class C), and *Arabidopsis *Hsf sequences At4g17750 (class A), At4g36990 (class B), and At5g62020 (class C). Second, DNATOOLS software was used to build local databases from the maize complete genome nucleotide sequences and protein sequences. The Hsf domain numbered PF00447 obtained from the Pfam database was used as a standard sequence to isolate all possible homologs in maize by BLASTP searches (*P*-value = 0.001). This step was crucial to identify as many similar sequences as possible. Moreover, the starting locations of all candidate Hsf genes on each chromosome were acquired by TBLASTN (*P*-value = 0.001). Through this method, the physical locations of all candidate Hsf genes were confirmed and the redundant sequences with the same chromosome location were rejected from the Hsf candidate list. Furthermore, all candidate sequences that met the standards were analyzed in the Pfam database once more and were detected by the SMART program [38] for the purposes of eliminating any sequences not containing the Hsf-type DBD domain. Finally, the remaining sequences were checked by means of the SMART program to recognize coiled-coil structure, which is the core of the HR-A/B region. The sequences without coiled-coil structure were removed. A distinctive name for each of Hsfs identified in maize was given according to its position from the top to the bottom on the maize chromosomes 1 to 10. Finally, the chromosome location image of Hsf genes was generated by MapInspect software [39].

### Multiple sequence alignment and domain prediction

Initially, ClustalX (version 1.83) [40] was performed to align amino acid sequences of Hsf proteins, which passed screening and were accepted. Subsequently, GeneDoc was used to manually edit the results. The domain analysis programs MARCOIL [41], PredictNLS [42] and NetNES 1.1 [43] were suitable for predicting coiled-coil domains, NLS and NES, respectively encoding Hsf genes. Additionally, the Hsf protein conserved motifs were defined by submitting their full-length amino acid sequences to MEME [44].

### Analysis of phylogenetic relationships and gene duplication

Phylogenetic trees were constructed by the neighbor-joining (NJ) method in MEGA (version 4.0) [45]. NJ analysis was performed with the Pairwise Deletion option and the Possion correction. For statistical reliability, bootstrap analysis was conducted with 1,000 replicates to assess statistical support for each node. *S. cerevisiae *Hsf1 (ScHsf1) was used as the outgroup.

Hsf gene duplication events were also investigated. MEGA (version 4.0) was used to align Hsf amino acid sequences by Clustal W and compute their evolutionary distances [46]. We defined a gene duplication according to the following criteria [46,47]: (1) the length of alignable sequence cover > 80% of the longer gene; and (2) the similarity of the aligned regions > 70%.

### Digital expression analysis: EST expression profile

The analysis of *ZmHsfs *expression profiles was accomplished by searching the maize dbEST database and finding expression information provided at the Web sites. Maize expression data was first obtained through blast searches against the maize dbEST database downloaded from NCBI by conducting the DNATOOLS Blast program. Searching parameters were as followings: maximum identity > 95%, length > 200 bp and Evalue < 10^-10^. In addition to the maize EST database, maize expression data was also extracted from the Maize Assembled Genomic Island (MAGI) [48] and the Plant Genomic Database (PlantGBD) [49] including EST, cDNA and PUTs (PlantGDB unique transcripts).

### Plant materials and stress treatment

Maize (*Zea mays *L. inbred line B73) plants were grown in a greenhouse at 28 ± 2°C with a photoperiod of 14 h light and 10 h dark. For heat stress, uniform-sized seedlings were transferred to a growth chamber to 42 ± 1°C when they developed three fully opened trifoliate leaves (approximately three weeks after sowing). The leaves of the seedlings were harvested after 0 and 1 h of heat stress treatment, frozen immediately in liquid nitrogen, and stored at -80°C until RNA isolation.

### RNA isolation and quantitative real-time PCR (qRT-PCR) analyses

To confirm the expression of representative of *ZmHsf *genes, total RNA was prepared using Trizol reagent (Invitrogen, USA), followed by DNase I treatment to remove any genomic DNA contamination. RNA concentration was determined by NanoDrop ND-1000 UV-Vis spectrophotometer (NanoDrop Technologies, Inc.) and the integrity of the RNA was assessed on a 1% (w/v) agarose gel. The first-strand cDNA was synthesized from 1 μg of total RNA using QuantiTect Rev. Transcription Kit (Qiagen, Germany). Quantitative RT-PCR was carried out using an ABI PRISM 7300 real-time PCR system (Applied Biosystems, USA). Each reaction contains 10 μL 2×SYBR Green Master Mix Reagent (Applied Biosystems, USA), 2.0 μL cDNA sample, and 400 nM of gene-specific primer in a final volume of 20 μL. Each pair of primers were designed by using Primer Express 3.0 software (Applied Biosystems, USA) targeting an amplicon size of 90-190 bp. The primers used are listed in the additional file 1. The thermal cycle used was as follows: 50°C for 2 min, 95°C for 10 min, 40 cycles of 95°C for 15 s, and 60°C for 1 min. The specificity of the reactions was verified by melting curve analysis. The relative mRNA level for each gene was calculated as ΔΔ*C*_T _values in comparison to unstressed seedlings (Applied Biosystems, USA). Maize *Actin 1 *gene was used as internal control for normalization. At least three replicates of each cDNA sample were performed for quantitative RT-PCR analysis.

## List of Abbreviations

AHA: activator motif; CTAD: C-terminal activation domain; DBD: DNA-binding domain; HS: heat shock; HSEs: heat shock elements; Hsfs: heat shock transcription factors; NES: nuclear export signal; NLS: nuclear localization signal; qRT-PCR: quantitative real-time PCR.

## Authors' contributions

These studies were designed by YXL, HYJ and BJC. YXL carried out all the experimental analyses and prepared all figures and tables. YXL and HYJ analyzed the data and drafted the manuscript. ZXC and XLT contributed to revisions of the manuscript. BJC and SWZ assisted in explaining the results and revised the final version of the manuscript. All authors have read and approved the final manuscript.

## Supplementary Material

Additional file 1 **Primers used in quantitative real-time PCR**. Excel document contains two tables listing primer sequences used for quantitative real-time PCR to validate expression patterns of *ZmHsf *genes. Click here for file
